# Knowledge and competences in hematological malignancies amongst radiation oncology residents in Germany—results from a national survey

**DOI:** 10.1007/s00066-024-02236-4

**Published:** 2024-04-29

**Authors:** Stephan Rehn, Michael Oertel, Philipp Linde, Matthias Mäurer, Khaled Elsayad, Niklas B. Pepper, Daniel Rolf, Jenna M. Kahn, John P. Plastaras, Jillian R. Gunther, Hans T. Eich

**Affiliations:** 1 Department of Radiation Oncology, University Hospital Muenster, Albert-Schweitzer-Campus 1, building A1, 48149 Muenster, Germany; 2 Department of Radiation Oncology, Cyberknife and Radiation Therapy, University Hospital of Cologne, Cologne, Germany; 3 Department for Radiotherapy and Radiation Oncology, University Hospital Jena, Jena, Germany; 4 Department of Radiation Medicine, Oregon Health and Science University, Portland, OR USA; 5 Department of Radiation Oncology, University of Pennsylvania, Philadelphia, PA USA; 6 Department of Radiation Oncology, The University of Texas MD Anderson Cancer Center, Houston, TX USA

**Keywords:** Medical education, Radiation therapy, Lymphoma, Leukemia, Competence-based education

## Abstract

**Introduction:**

Radiation oncology is a pivotal modality in the treatment of hematologic malignancies. To enable state-of-the-art patient care, structured education during residency is essential. However, given the lack of detailed data, the scope of educational opportunities available to trainees remains elusive. This prompted our group to perform a national survey amongst radiation oncology residents in Germany assessing the status quo of competences in the treatment of lymphoma and leukemia patients. Furthermore, areas of potential improvement were identified to further the goal of competence-based education for residents.

**Methods:**

A survey-based analysis was conducted to assess the knowledge and competence of radiation oncology residents in Germany regarding hematological malignancies. A decisive questionnaire covering demographics, self-assessment of competences, and areas for improvement was developed in adaption of a survey by the Association of Residents in Radiation Oncology and distributed amongst 1439 members of the German Society of Radiation Oncology. Responses were collected anonymously via an online survey tool and analyzed using descriptive statistics and chi-square tests.

**Results:**

A total of 59 complete and 22 partial responses were collected, yielding a 5.6% response rate. Participants’ competence varied, with notable experience gaps in pediatric cases, proton therapy, and large-field techniques like total-skin irradiation or pediatric total body irradiation. While participants felt confident in treatment planning and patient counseling, they showed deficiencies in the definition of the planning target volume for modern involved site radiotherapy. Resources for education included national and international guidelines, scientific reviews, and textbooks. Board-certified radiation oncologists and physicians from specialized lymphoma centers demonstrated higher overall competence levels.

**Conclusion:**

This survey highlights the diversity of resident education regarding hematological malignancies in German radiation oncology programs. Knowledge gaps exist in key areas, including pediatric cases and specialized techniques. Competence-based education, interactive teaching formats, and rotations to specialized centers are potential strategies to address these gaps. The study contributes to the understanding of the federal educational landscape, underscoring the need for standardized and comprehensive training to ensure optimal patient care in hematological malignancies within the context of radiation oncology. Further research and collaborations are warranted to enhance training and expertise in this critical domain.

## Introduction

Continued medical education during residency is a key factor to enable formation of a professional identity [1]. With approximately 1500 practicing board-certified physicians in Germany, radiation oncology (RO) represents a small but important clinical discipline as around 50% of oncological patients undergo radiation treatment during the course of their disease [2, 3]. The educational situation is complicated by the federal organization in Germany, which includes different teaching concepts being in action [4, 5]. However, a general curriculum has been defined by the German Society of Radiation Oncology (DEGRO) including mandatory areas of expertise and competence to ensure standards for board certification [6, 7]. Hematological malignancies like lymphoma and leukemia represent particularly challenging diseases, encompassing > 100 different entities in the current issue of the World Health Organization manual [8]. In the DEGRO curriculum, leukemia, Hodgkin, and non-Hodgkin lymphoma as well as multiple myeloma are mentioned, covering a wide area of treatment scenarios [6]. Furthermore, recent clinical guidelines advocate for the use of RO for hematological malignancies even in situations of critical shortage of resources [9, 10]. Nevertheless, hematologic neoplasia are only covered in approximately 40% of all RO curricula in medical schools, as shown by a survey of the working group youngDEGRO [11], which may lead to a knowledge gap for future residents. This is mirrored by another survey of the youngDEGRO amongst RO residents, in which the specified level of knowledge for leukemia and lymphoma was mostly “bad to mediocre” [4]. A detailed assessment of expertise on hematologic malignancies and the ability to treat them has been performed by the Association of Residents in RO (ARRO) in the United States and depicts a “moderate” overall preparation [12]. A similar survey does not exist for the resident formation in Europe or Germany. Therefore, our group decided to perform a detailed analysis on RO resident training for hematological malignancies, investigating skills and knowledge. It thereby reflects strengths and potential fields of improvements and points towards further deepening of competence-based education for residents.

## Materials and methods

### Questionnaire

A 17-part questionnaire was developed in a multistep peer-review process by the authors of the current manuscript in adaption of a survey conducted by the ARRO which was published in February 2023 [12]. The current questionnaire encompasses demographic information, questions about self-assessment of competences in treatment techniques, radiation planning and contouring, as well as possible opportunities for further optimization. Different types of questions were used, such as Likert-type scale questions with a rating scale from 1 (not at all) to 5 (extremely/very good), binary questions, and multiple-choice questions, with some of them offering the opportunity to provide a free-text commentary. The survey was designed using the online survey tool LimeSurvey version 5.5.0 (LimeSurvey GmbH, Hamburg, Germany) and distributed anonymously via email amongst DEGRO members. A total of 1439 members were contacted on November 15, 2022, and December 22, 2022.

### Statistical analysis

Results were exported to Microsoft Excel (Microsoft, Redmond, WA, USA) and summarized via absolute numbers and percentages. Further statistical analyses were done using SPSS version 29 (IBM, Armonk, NY, USA): for testing between categorical variables, a chi-square test was used. All tests were regarded as statistically significant with a *p*-value of 0.05 or below.

## Results

The survey was completed by 59 individuals in full and 22 partially, which thus corresponds to a total response rate of 5.6%. Both partial and full responses were included in the analysis and numbers were adjusted accordingly.

### Demographics

The survey participants were evenly distributed by gender (47.2% male, 51.4% female, 1.4% no disclosure) and mostly in their second to fifth year of residency (cumulative 58.6%); 35.7% were already board-certified specialists (Table 1). Participants predominantly worked in an institution with 5–8 residents (31.8%), followed by 1–4 residents (28.8%), 9–12 residents (21.2%), 17 residents or more (10.6%), and 13–16 residents (7.6%). The majority reported treating patients with leukemia and lymphoma at their institution on a regular basis (90.1%).

**Table 1:** Demographics and characteristics of participants

Participant demographics and characteristics	*N*	%
*Sex*		
Male	34	47.2
Female	37	51.4
Not specified	1	1.4
*Age (years)*		
20–24	0	0.0
25–29	15	20.0
30–35	33	44.0
36 or older	27	36.0
*Duration of residency (in years)*		
1	1	1.4
2	9	12.9
3	12	17.1
4	8	11.4
5	12	17.1
> 5	3	4.3
Specialist	25	35.7
*Number of residents in own institution*		
1–4	19	28.8
5–8	21	31.8
9–12	14	21.2
13–16	5	7.6
17 or more	7	10.6
*Leukemia and lymphoma treatment on a regular basis*		
Yes	64	90.1
No	7	9.9

### Self-reported experiences and competences

**Fig. 1:**
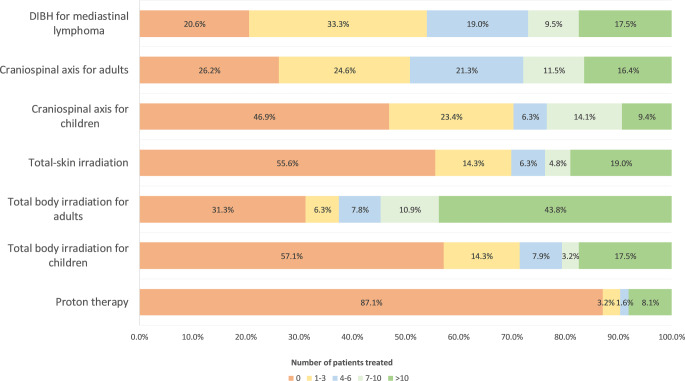
Experience with radiation therapy techniques of the participants. *DIBH *deep-inspiration breath-hold

Participants reported a total lack of experience (i.e., zero cases) for irradiation of a craniospinal axis in children (46.9%), total skin irradiation (55.6%), total body irradiation (TBI) prior to allogeneic stem cell transplantation in children (57.1%), and proton therapy (87.1%; Fig. 1). In contrast, participants claimed greater experience (7 or more patients treated) with TBI for adults (54.7%), deep-inspiration breath-hold technique for mediastinal lymphoma (27.0%), and craniospinal axis for adults (27.9%).

**Fig. 2:**
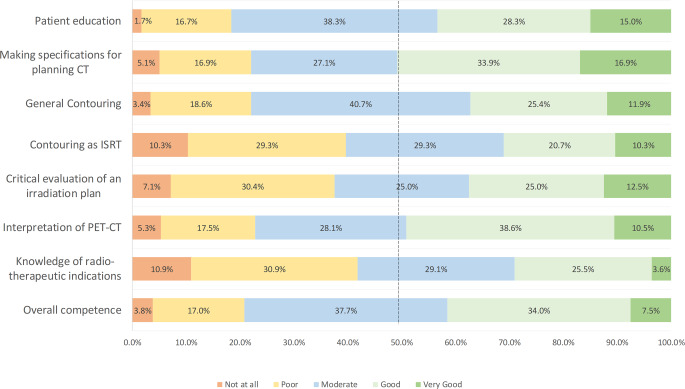
Level of competence. *CT* computed tomography, *ISRT* involved site radiotherapy, *PET* positron-emission tomography

Participants felt most confident in making specifications for the planning computed tomography (CT) in lymphoma radiation therapy, with 50.8% reporting good or very good confidence. When asked about their readiness for patient counseling/education, 15.0% and 28.3% responded to have very good or good competences, respectively, compared to 38.3% with moderate and 16.7% with poor competences (1.7% responded “not at all,” Fig. 2). The majority had moderate, poor, or no proficiency in defining the planning target volume for modern involved site radiotherapy (ISRT; 69.0% vs. 31.0% for good to very good proficiency). General competence in contouring of lymphoma (without explicit contouring specification of ISRT) was evaluated to be better: 11.9% reported very good, 25.4% good, 40.7% moderate, 18.6% poor, and 3.4% no experience. In terms of critical evaluation of an irradiation plan (technique, field arrangement, organs at risk), the participants’ answers were well balanced, with 12.5% reporting very good, 25.0% good, 25.0% moderate, 30.4% poor, and 7.1% no experience at all. Resources being used by participants for ongoing education and training in the treatment of lymphomas/leukemia were national guideline(s) (61.7%), international guideline(s) (54.3%), scientific reviews (34.6%), conference presentations (written or via video; 18.5%), podcasts (1.2%), textbooks (40.7%), and online sources (43.2%). The overall competence to treat patients with lymphoma and/or leukemia was reported as very good by 7.5%, good by 34.0%, moderate by 37.7%, poor by 17.0%, and non-existent by 3.8%. Overall, higher levels of competences for the treatment of lymphoma in general were described by board-certified radiation oncologists (*p* = 0.049) and physicians from specialized centers with a focus on lymphoma treatment (*p* < 0.001) in comparison to all other respondents, respectively. In contrast, age and the number of residents in one’s own institution had no significant impact on the self-reported overall competence (*p* = 0.392 and *p* = 0.257, respectively).

### Opportunities for improvement

Regarding suggestions for optimization of the medical training in hematological malignancies, participants mentioned having more symposia/seminars in the annual meeting program (13.5%), training events involving “frontal teaching” such as seminars or lectures (38.2%), more online resources (i.e., podcasts, lectures, recordings; 33.3%), interactive courses (51.8%), and a rotation to dedicated specialized centers (34.6%), respectively.

## Discussion

The conducted survey reflects the complex and heterogeneous condition of resident formation in Germany concerning hematological malignancies. It demonstrates that 1) the majority report an overall competence to treat hematological malignancies which is good to moderate, but this assessment differs remarkably between the participants; 2) although most participants claim that their hospital/private practice treats lymphoma and leukemia patients on a regular basis, patient numbers are low, and that 3) this is especially true for pediatric patients and special techniques like total-skin irradiation and proton treatment; 4) there is an unmet need for more competence-based and interactive teaching formats.

Comparing the German participants’ experience with those of the American ARRO survey reveals similarities and differences. Whereas the German participants reported a lack of experience with proton therapy in 87.1% of the cases, only 68.6% of the ARRO participants did. Particularly, 30.3% of the American participants even reported a patient number treated with protons exceeding 10, suggesting that proton therapy plays a greater role in the United States, both in treatment and in resident training. Apart from that, the German participants claimed to be better prepared or to have a higher level of experience concerning many aspects of hematologic malignancies: 27.9% of the German participants reported a higher number of adult patients ( 7 or more) treated with craniospinal axis vs. 8.7% of the American participants. Overall, 41.5% of the German participants felt well or very well prepared for the treatment of leukemia and lymphoma, compared with only 26.9% of the ARRO participants.

Although less common than solid tumors, lymphoma and leukemia are still an integral part of the standard curriculum for board certification in RO, both in the present [6] and in the upcoming new licensing regulations [13]. The latter represents a shift from mere knowledge acquisition to competence-based education focusing on practical skills. This evolution challenges established teaching practices and aims to involve students (and latter residents) more actively. Concerning RO, examples of new teaching formats have been presented which are establishing a link to basic subjects (anatomy, physiology) [14], deepening the understanding of brachytherapy [15] or introducing digital concepts [16]. Correspondingly, Linde et al. elaborated a desire for practical education and early integration of RO in the curriculum amongst medical students [17]. Knowledge should be deepened and extended in the respective specialty areas during resident formation. However, data from surveys conducted by the youngDEGRO suggest that hematological malignancies are only taught in a minority of medical faculties (41.7%), with a resulting bad to mediocre resident knowledge [4, 11].

A particular lack of expertise is evident for pediatric patients, as 46.9% and 57.1% of all contributors have never participated in the treatment of a craniospinal irradiation or TBI in children, respectively. The latter figure is worrisome, as TBI was shown to be a superior conditioning modality for pediatric patients with acute lymphoblastic leukemia in comparison to a chemotherapy-only strategy in the randomized FORUM trial [18]. At the same time, pediatric patients are vulnerable to radiation effects, with an increased rate of secondary malignancies after TBI [19]. Thus, critical evaluation and discussion of both the efficacy and toxicity of radiation treatment (by well-trained board-certified radiation oncologists) is needed. Likewise, some techniques like proton therapy are only available at sparse specialized centers, explaining the lack of expertise here.

Only 30–50% of participants reported feeling that they have good or very good knowledge of the correct specification of planning CT and target volume definition. Interestingly, reported confidence numbers declined when contouring according to the principles of ISRT is demanded in comparison to a contouring in general. This suggests that at least some participants tend to define target volumes for lymphoma in a non-structured way. This is surprising in the light of several contouring and treatment guidelines provided by the International Lymphoma Radiation Oncology Group [20–23].

The survey conducted has several limitations. Due to the voluntary participation, the response rate is incomplete. Furthermore, it is likely that particularly motivated residents or those working at a specialized center were more likely to respond. Considering this selection bias, the overall situation is likely worse, and the results may not be representative for the federal situation. To increase the participation rate, future surveys should be conducted on multiple platforms, also including social media, and should be highlighted with reminders/direct links to the questionnaire during the annual meeting of DEGRO. Additionally, the questionnaire relied on self-assessment only, without asking for external assessment by those responsible for training organization (senior physicians, head of departments) or considering objective outcomes (results of board certification, scientific abstracts, or papers on hematological malignancies).

Nevertheless, the data presented are a valuable addition to the overall picture of the educational situation in Germany. As only two national societies in radiation oncology have surveyed their hematological resident formation so far, more evaluations will have to be performed for a global or at least multinational perspective.

Future developments must address resident formation on multiple levels. Interactive workshops, digital courses, or tumor boards may help to broaden knowledge and practical treatment skills for lymphoma. To enable sufficient patient numbers for these entities, a rotation program may be useful; this has already been implemented by the youngDEGRO. As participant numbers increase after the COVID-19 pandemic, evaluation results of this effort are eagerly awaited. Together with the reference radiation oncologists of the German Lymphoma Alliance and German Hodgkin Study Group, these efforts will aim to broaden and deepen resident knowledge of modern radiation oncology treatment for leukemia and lymphoma. In the end, generating and maintaining radiation oncology expertise in hematological malignancies will be essential to develop personalized treatment strategies for the individual patient.
